# Complete genome and phylogenetic characterization of a novel papillomavirus from *Cuniculus paca* in the Brazilian Amazon

**DOI:** 10.1007/s00705-026-06636-y

**Published:** 2026-05-07

**Authors:** Cleyton Silva de Araújo, Laura Gabrielly Alves Henrique, Hanna Gabriela da Silva Oliveira, Jhonatan Henrique Lima da Rocha, Junio Damasceno de Souza¹, Agnes de Souza Lima, Vania Maria França Ribeiro, Francisco Carlos da Silva, David Driemeier, Cláudio Wageck Canal, John Munday, Felipe Masiero Salvarani, Flavio Roberto Chaves da Silva, Cíntia Daudt

**Affiliations:** 1 Laboratório de Virologia Geral e Parasitologia, Centro de Ciências Biológicas e da Natureza, Universidade Federal do Acre, Campus Universitário, BR 364, Km 04-Distrito Industrial, Rio Branco, 69920-900 AC Brazil; 2 Laboratório de Medicina Veterinária Preventiva e Vacinologia, Instituto de Medicina Veterinária, Universidade Federal do Pará, Castanhal, 68740-970 PA Brazil; 3 Setor de Patologia, Faculdade de Veterinária, Universidade Federal do Rio Grande do Sul, Av. Bento Gonçalves, Prédio 42.505, Porto Alegre, 9090, 91540-000 RS Brazil; 4 Laboratório de Virologia, Faculdade de Veterinária, Universidade Federal do Rio Grande do Sul, Av. Bento Gonçalves, Prédio 42.505, Porto Alegre, 9090, 91540-000 RS Brazil; 5 School of Veterinary Science, Massey University, Palmerston North, 4410 New Zealand

**Keywords:** *Papillomaviridae*, *Cuniculus paca*, Rodent virus, Genome characterization, Amazon biome, Phylogeny

## Abstract

Wild rodents remain underrepresented in papillomavirus genomics, limiting current understanding of host range, tissue tropism, and evolutionary diversity. Here, we report the first papillomavirus identified in the spotted lowland paca, *Cuniculus paca*, from the Brazilian Amazon, provisionally designated *Cuniculus paca* papillomavirus 1 (CpPV1). Histopathological examination supported the diagnosis of a virally induced squamous papilloma. Broad-range L1 PCR (FAP59/64), rolling-circle amplification, and Illumina MiSeq sequencing enabled recovery of a complete circular genome of 8,803 bp containing the canonical early (E6, E7, E1, and E2) and late (L2 and L1) open reading frames, together with a typical long control region. Pairwise comparison of the complete L1 gene showed 63.10% nucleotide identity to *Erethizon dorsatum* papillomavirus 2 (EdPV2), well below the threshold used for papillomavirus type demarcation. Maximum-likelihood phylogenetic analysis recovered CpPV1 as a distinct lineage sister to EdPV2 within a broader, non-monophyletic rodent papillomavirus assemblage. This finding expands the known diversity of *Papillomaviridae* in Neotropical rodents and provides new insight into papillomavirus evolution in an under-sampled host group.

**Graphical Abstract:**

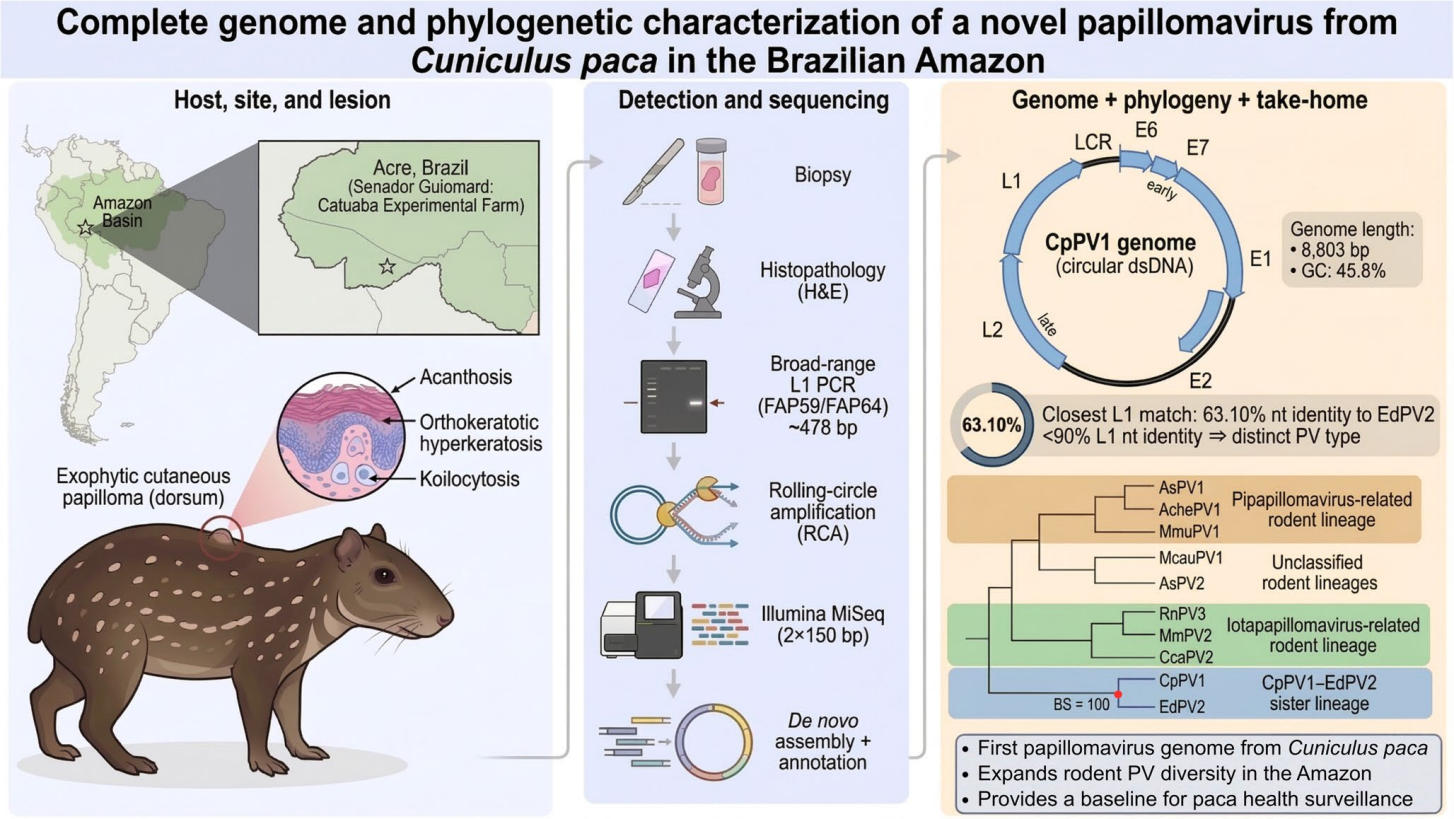

**Supplementary Information:**

The online version contains supplementary material available at 10.1007/s00705-026-06636-y.

## Introduction

Papillomaviruses (PV) are small, non-enveloped, double-stranded DNA viruses within the *Papillomaviridae* family, characterized by strict host specificity and a marked tropism for cutaneous and mucosal epithelia across a broad range of vertebrates, including humans, domestic animals, and wildlife [1, 2]. Although hundreds of human PV types have been fully characterized, PV diversity in non-human hosts especially wild rodents remains markedly underexplored [1, 3, 4]. Most current insights into rodent PVs derive from a narrow set of taxa and experimental model systems [3, 5–9], leaving the natural diversity, host range, and evolutionary placement of rodent PVs in free-living species poorly resolved.

In global biodiversity hotspots with complex multi-host assemblages, such as the Amazon rainforest, there is likely a substantial and largely unsampled reservoir of non-human PVs [10]. Rodents are abundant, ecologically plastic, and frequent users of human-modified landscapes, making them informative sentinels for mapping viral diversity and host associations in situ [11]. Yet, within Neotropical hystricomorph rodents, molecular data on papillomaviruses are virtually absent.

The spotted paca (*Cuniculus paca* Linnaeus, 1766) is a medium-sized hystricomorph rodent widely distributed throughout the Neotropics, occupying both primary and secondary forests as well as human-altered habitats, and holds socioeconomic relevance as a managed game species; however, wild populations face pressure from illegal hunting [12]. Despite this ecological and economic importance, papillomaviruses from *C. paca* have not been molecularly characterized, and their phylogenetic relationships to known rodent PVs are unknown. This represents a specific gap in our understanding of Amazonian wildlife health and of PV diversification in Hystricomorpha.

Here, we report the detection and whole-genome characterization of a novel papillomavirus from a free-living *C. paca* in the Amazon biome, provisionally designated *Cuniculus paca* papillomavirus 1 (CpPV1), identified following the observation of a papillomatous skin lesion. By integrating histopathology, broad-range L1 PCR, genome recovery and annotation, and phylogenomics, our objectives were to (i) detect and characterize PV DNA in lesion tissue, (ii) recover and annotate the complete viral genome, (iii) infer its phylogenetic placement and taxonomic status relative to established PV types, and (iv) document histopathological features consistent with PV infection in this host. Collectively, these analyses characterize CpPV1 as a previously unrecognized PV type infecting a Neotropical hystricomorph rodent and expand the known host spectrum of *Papillomaviridae* in South America.

## Materials and methods

### Ethics statement

All procedures complied with the guidelines of the Brazilian College of Animal Experimentation (COBEA) and were approved by the Ethics Committee on Animal Use at the Federal University of Acre (Protocol No. 23107.005499/2018-96).

### Sample collection

**Fig. 1:**
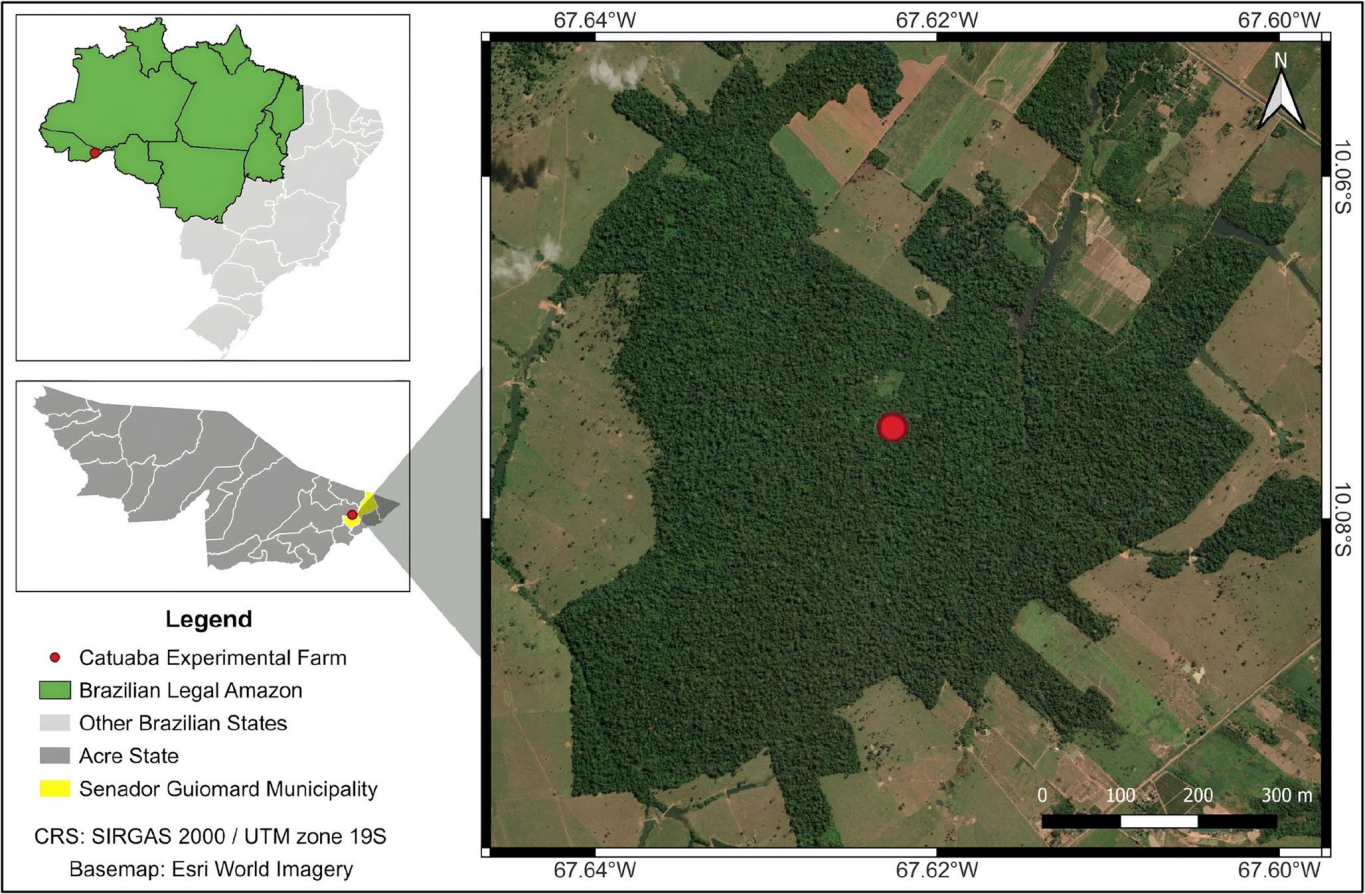
Location of the study site in the Brazilian legal Amazon. The study was conducted at the Catuaba experimental farm, Federal University of Acre, in Senador Guiomard, Acre, Brazil (10°04′20.5″S, 67°37′38.7″W). Coordinate reference system (CRS): SIRGAS 2000 / UTM zone 19 S. Basemap: Esri World Imagery

A free-living *Cuniculus paca* presenting a single pedunculated, wart-like skin mass (0.5 cm in diameter) on the dorsal region was identified within a breeding cohort maintained at the Catuaba Experimental Farm of the Federal University of Acre, municipality of Senador Guiomard, Acre State, Brazil (10°04′20.5″S, 67°37′38.7″W; Fig. 1). Following capture by net restraint, local anesthesia was achieved with 2% lidocaine (Bravet, Brazil), and the lesion was removed by excisional biopsy using sterile scalpels and forceps. The specimen was bisected: one portion was fixed in 10% neutral-buffered formalin for histopathological examination, and the other was stored at − 20 °C pending DNA extraction.

### Histopathology

Fixed tissue was processed after a minimum of 72 h in 10% neutral-buffered formalin. Sections were routinely embedded in paraffin, cut, and stained with hematoxylin and eosin (H&E) for light-microscopic examination.

### DNA extraction, PCR and Sequencing

Total DNA was extracted from frozen papilloma tissue using the PureLink^®^ Genomic DNA Mini Kit (Invitrogen, Carlsbad, CA, USA) according to the manufacturer’s instructions. Partial amplification of the L1 gene was carried out with the degenerate primer pair FAP59 (5′-TAA CWG TIG GIC AYC CWT ATT-3′; BPV1 × 02346 positions 5712–5752) and FAP64 (5′-CCW ATA TCW VHC ATI TCI CCA TC-3′; BPV1 × 02346 positions 6185–6206), yielding an expected amplicon of approximately 478 bp [13]. PCR was performed as previously described [14] in a 25 µL reaction containing 1× PCR buffer, 0.38 mM MgCl₂, 1 U Taq DNA polymerase, 0.05 mM each dNTP, 0.2 µM each primer, and 2 µL template DNA; nuclease-free water served as a no-template control. Thermal cycling was performed on a SimpliAmp™ Thermal Cycler (Applied Biosystems, Thermo Fisher Scientific) under the following conditions: initial denaturation at 94 °C for 2 min; 40 cycles of 94 °C for 1 min, 50 °C for 1 min, and 72 °C for 1 min; and a final extension at 72 °C for 7 min. Amplicons were visualized on a 2% agarose gel stained with Blue Green Loading Dye I (LGC, Brazil) under UV illumination.

The positive amplicon was purified with the NucleoSpin Extract II Kit (Macherey–Nagel, Düren, Germany) and sequenced bidirectionally on an ABI PRISM 3100 Genetic Analyzer (Applied Biosystems) using the BigDye Terminator v3.1 Cycle Sequencing Kit (Applied Biosystems). Resulting sequences were trimmed, assembled, and edited in Geneious Prime^®^ v2025.0.2.

### Rolling circle amplification and high throughput sequencing

The complete PV genome was enriched by rolling circle amplification (RCA) of circular viral DNA [15], using 100 ng of purified papilloma DNA as input with random hexamer primers. The RCA product was visualized on a 1% agarose gel, then purified using the Genomic DNA Clean & Concentrator kit (Zymo Research) following the manufacturer’s protocol. DNA purity and concentration were assessed by NanoDrop spectrophotometry (Thermo Scientific) and Qubit fluorometry (Invitrogen) prior to library preparation. Sequencing libraries were constructed from 1 ng of input DNA using the Nextera XT DNA Library Preparation Kit (Illumina) and sequenced on an Illumina MiSeq platform (2 × 150 bp paired-end reads, v2 chemistry).

### Genome assemblies and sequence analyses

Raw paired-end reads were quality-checked and trimmed to remove adapters and low-quality bases prior to de novo assembly. High-quality reads were assembled using SPAdes v3.15.3 with default parameters. The resulting contigs were screened against the NCBI GenBank nucleotide and protein databases using BLASTn and BLASTx, respectively, without restricting the search to papillomavirus sequences. Contigs of all lengths were initially evaluated, with particular attention to those exceeding 1,000 nt, as commonly reported in papillomavirus studies. Candidate viral contigs were subsequently inspected and curated manually. Genome annotation, including prediction of open reading frames (ORFs) and conserved domains, was performed in Geneious Prime^®^ v2025.2, with reference to previously characterized rodent papillomaviruses to support annotation accuracy.

ORFs were predicted and annotated in Geneious Prime^®^ by combining automated ORF prediction, conserved-domain inspection, motif-based searches, and manual curation. Annotation was refined by comparison with representative rodent papillomavirus genomes available in GenBank/PaVE. Conserved papillomavirus-associated motifs were then evaluated in the predicted proteins and in the non-coding regulatory region. Specifically, E6 and E7 were screened for the canonical cysteine-rich zinc-binding motif (C-x2-C-x29-C-x2-C); the class I C-terminal PDZ-binding motif was assessed in E6, and the retinoblastoma protein-binding motif (L-x-C-x-E) in E7. In E1, we searched for conserved helicase-related signatures, including the Walker A/P-loop ATP-binding motif, a Walker B-like acidic motif, and a putative Sensor 1-like motif. In E2, the presence of a leucine zipper-like motif was evaluated. In L2, we screened for a canonical N-terminal furin-cleavage motif, glycine-rich G-x3-G motifs in the N-terminal region, a putative retromer-binding FYL-like motif, and a C-terminal Lys/Arg-rich basic region. L1 was similarly assessed for a C-terminal Lys/Arg-rich basic tail. The long control region (LCR/URR) was examined for canonical regulatory elements, including E2-binding sites, E1 recognition half-sites, and polyadenylation signals. Motif coordinates were recorded as amino acid intervals for coding regions and as nucleotide positions for non-coding regions. No E5 ORF was identified.

### Comparative dataset: targeted review of rodent papillomaviruses (PaVE/GenBank/ICTV)

A comparative dataset was compiled to contextualize CpPV1 within known rodent PV diversity. We queried PaVE (pave.niaid.nih.gov; “Animal reference genomes”), GenBank/RefSeq (NCBI), and ICTV taxonomy reports, screening the primary descriptions of all rodent PV types. Records were included if they corresponded to order Rodentia and had a complete genome sequence publicly available as of August 2025. For each entry, we extracted host species, virus abbreviation, GenBank/RefSeq accession number, ICTV genus/species assignment (when available), tissue or lesion site of isolation (when reported), and country of first report. All data were compiled in a spreadsheet and cross-checked manually for consistency across sources.

### L1 dataset curation and pairwise identity

For inter-type comparisons, an L1 dataset was assembled comprising one representative sequence per rodent PV type identified above, together with the CpPV1 L1 sequence. Complete L1 nucleotide sequences were aligned in Geneious Prime^®^ using translation-guided MUSCLE alignment, followed by manual inspection and terminal trimming, with removal of gap-rich, poorly aligned regions, yielding a final alignment matrix of 1,494 nt. Pairwise nucleotide identity values were computed directly from the trimmed alignment in Geneious Prime^®^. Following the established PV type demarcation threshold, L1 nucleotide identity below 90% relative to the nearest recognized type was interpreted as evidence of a novel type. A similarity heatmap was generated from the resulting pairwise identity matrix for visualization.

### Phylogenetic Analysis

For the main phylogenetic analysis, a concatenated nucleotide dataset comprising the E1, E2, L2, and L1 coding regions was assembled from all complete rodent papillomavirus genomes, including CpPV1, for the main phylogenetic analysis. To provide broader family-level context, the publicly available ICTV *Papillomaviridae* reference backbone [16] a four-gene concatenated alignment of type-species isolates representing each PV genus was incorporated. This backbone was originally constructed from amino acid alignments (MUSCLE v7.221) followed by phylogenetic inference on the concatenated nucleotide sequences.

**Fig. 2:**
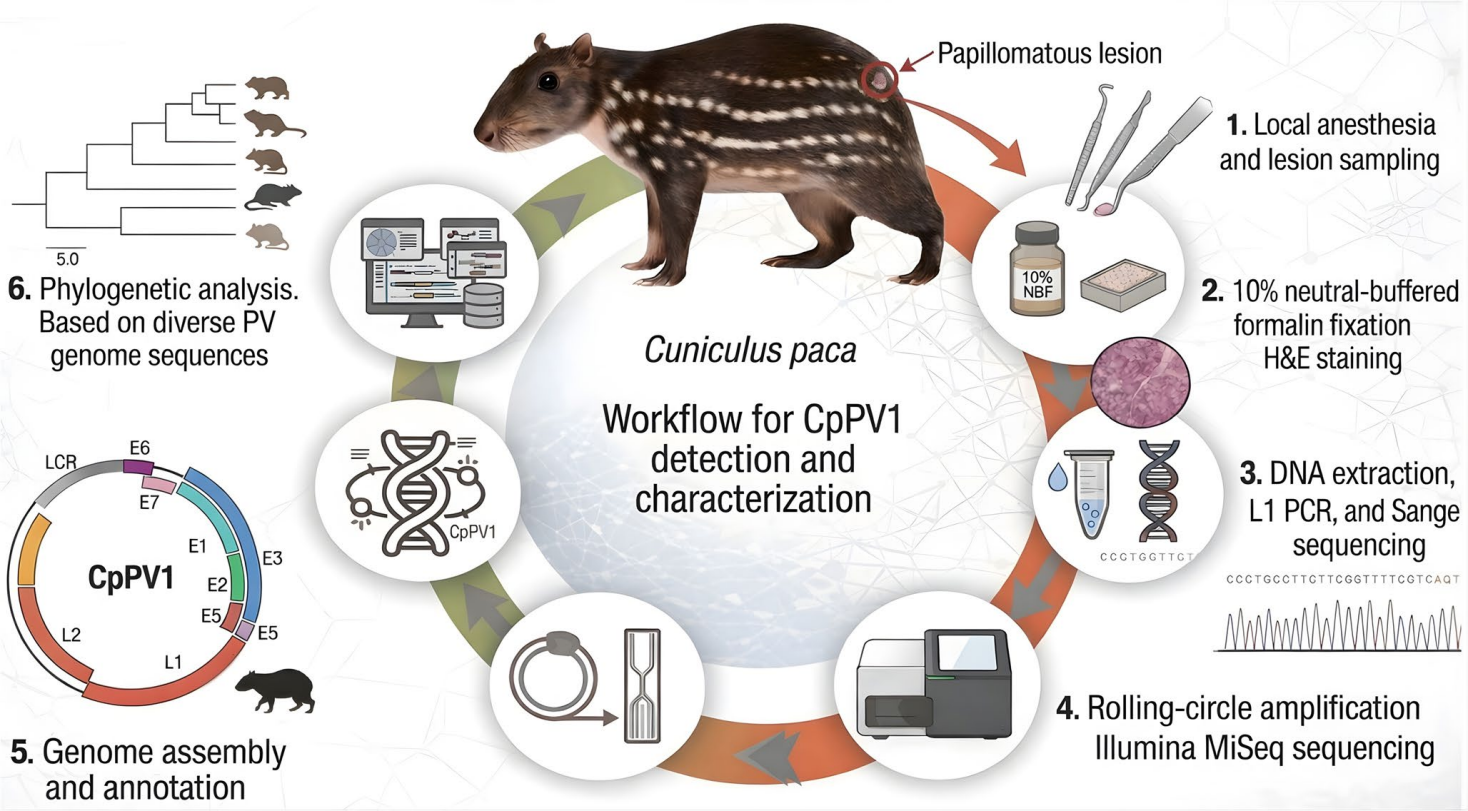
Workflow summarizing lesion sampling, histopathology, molecular screening, complete genome recovery, and phylogenetic characterization of CpPV1 from *Cuniculus paca*

Rodent PVs already represented in the ICTV backbone were retained from the reference dataset; those absent were integrated by extracting and concatenating the same four coding regions in the order E1–E2–L2–L1 and adding them to the pre-existing alignment using MAFFT v7, applying the add-to-existing-alignment strategy for full-length unaligned sequences while preserving the reference alignment structure. The augmented alignment was manually inspected prior to trimming and phylogenetic inference. Maximum-likelihood trees were inferred using the RAxML plugin in Geneious Prime^®^, with gene-partitioned analysis (E1, E2, L2, and L1), the General Time Reversible substitution model with gamma-distributed rate variation among sites (GTR + G), and 1,000 rapid bootstrap replicates. The overall analytical workflow is summarized in Fig. 2.

## Results

### Clinical presentation

**Fig. 3:**
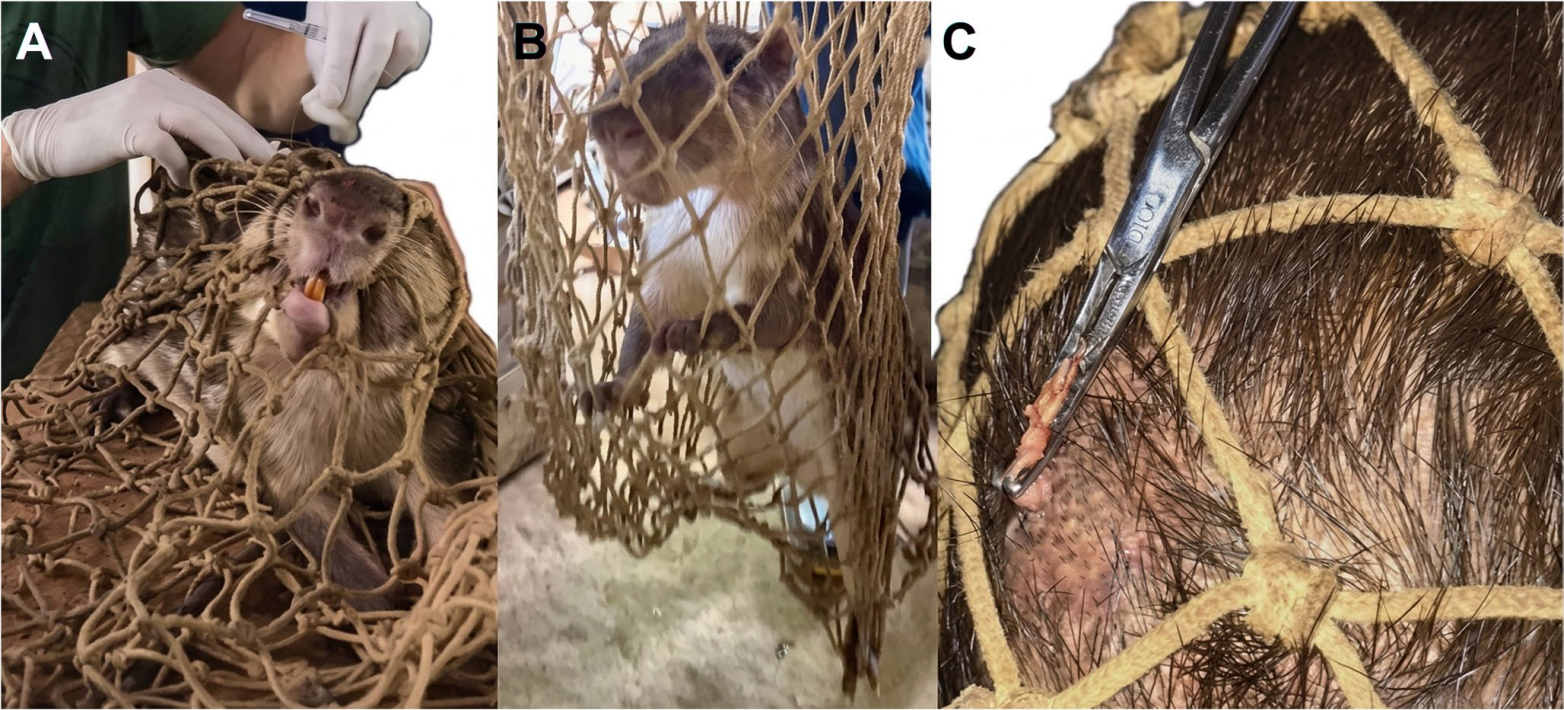
Clinical documentation of the paca examined in this study. (**A**, **B**) Manual restraint of *Cuniculus paca* using an appropriate capture net to allow clinical examination and collection of an excisional biopsy. (**C**) Exophytic papillomatous lesion located on the paca’s dorsal region

Physical examination following net restraint (Fig. 3A and B) revealed a single exophytic, pedunculated, wart-like mass located on the *dorsal region* of the animal (Fig. 3C).

### Histopathology

**Fig. 4:**
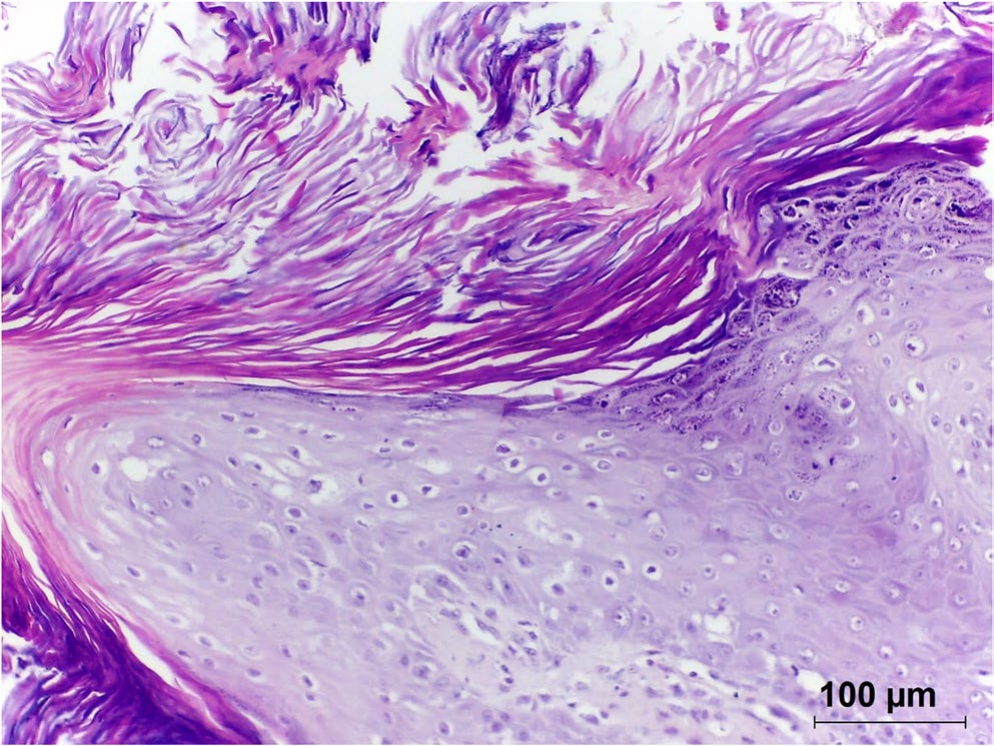
Exophytic wart-like mass on the skin of a *Cuniculus paca*. The lesion contains marked thickening and folding of the epidermis. The thickened epidermis is covered by increased quantities of compact orthokeratosis. Epidermal cells contain a dark shrunken nucleus surrounded by a clear halo, consistent with koilocytosis. Marked clumping of keratohyalin granules is visible within superficial layers of the thickened epidermis. The histological features are most consistent with a virally-induced squamous papilloma. HE 100X

Histopathological examination revealed epidermal proliferation of well-differentiated keratinocytes, with marked acanthosis, prominent keratohyalin granules in the granular layer, and orthokeratotic hyperkeratosis. Koilocytes enlarged keratinocytes with eccentric, pyknotic nuclei surrounded by a clear perinuclear halo were also identified (Fig. 4). These features were considered consistent with a virally induced squamous papilloma.

### L1 PCR detection

Degenerate primers FAP59/FAP64 amplified a ~ 478 bp L1 fragment from papilloma tissue, confirming the presence of PV DNA in the lesion. Sanger sequencing of the amplicon yielded high-quality bidirectional reads. BLASTn analysis indicated low nucleotide identity to all previously described rodent PVs, consistent with a divergent genotype.

### Genome recovery and features

Rolling circle amplification (RCA) followed by Illumina MiSeq sequencing and de novo assembly yielded a complete, circular papillomavirus genome of 8,803 bp, with a GC content of 45.8%, supported by 13,994 reads at a mean coverage of 272.9×. The genome was designated *Cuniculus paca* papillomavirus 1 (CpPV1) and deposited in GenBank under accession number PX891004.

**Fig. 5:**
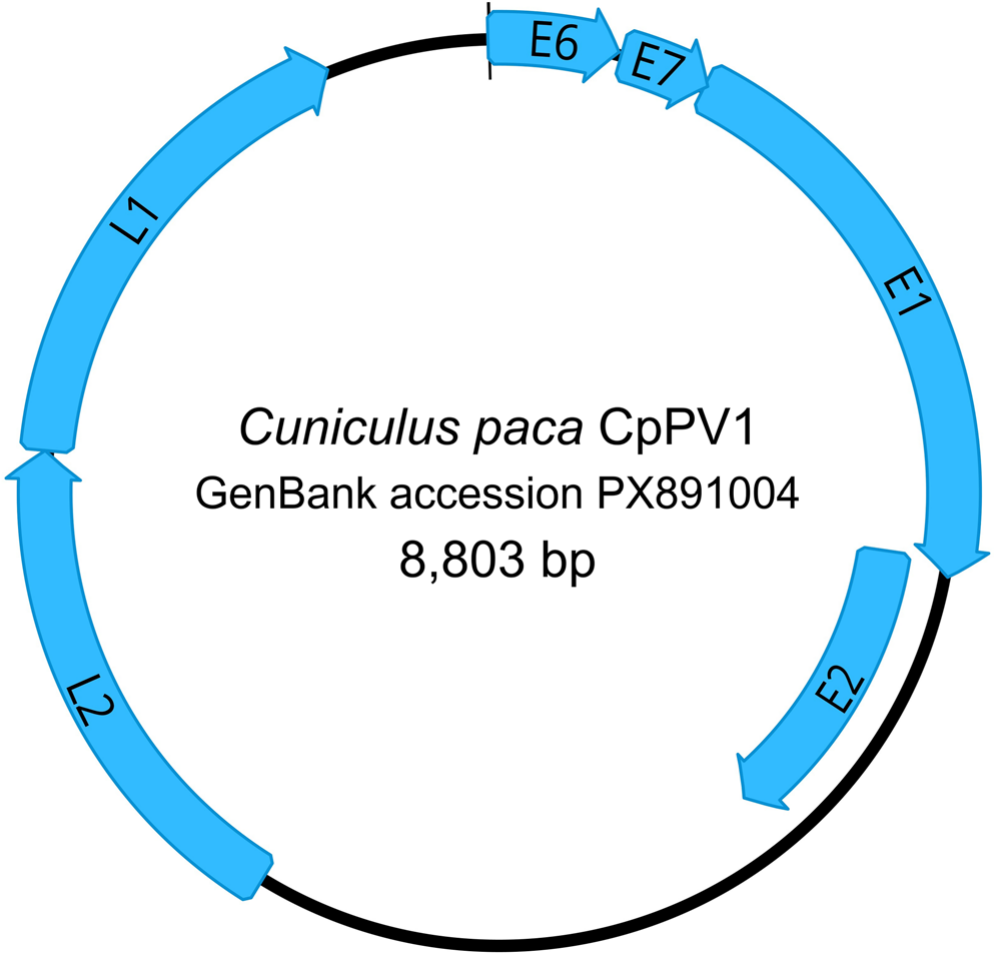
Genome organization of CpPV1 (GenBank accession PX891004) identified in this study. The first nucleotide was assigned within E6. CpPV1 contains the canonical early genes (E1, E2, E6, and E7), late genes (L1 and L2), and a non-coding long control region characteristic of papillomaviruses

The genome encodes the early ORFs E6, E7, E1, and E2, the late ORFs L1 and L2, and a non-coding long control region (LCR/URR) (Fig. 5). No E5 ORF was identified, consistent with the six-ORF genomic organization reported for many rodent papillomaviruses. Predicted ORF sizes were as follows: E6 (411 nt; 137 aa), E7 (282 nt; 94 aa), E1 (1,803 nt; 601 aa), E2 (1,047 nt; 349 aa), L2 (1,560 nt; 520 aa), and L1 (1,533 nt; 511 aa).

Motif screening supported canonical papillomavirus protein functions across all ORFs. E6 contained two conserved cysteine-rich zinc-binding motifs (C-x₂-C-x29-C-x₂-C), whereas a class I C-terminal PDZ-binding motif was absent. E7 retained a conserved zinc-binding motif but lacked both a canonical pRb-binding LxCxE motif and close variants thereof. In E1, conserved ATP-dependent helicase signatures were identified: the Walker A/P-loop motif (GPPDTGKS), a putative Walker B-like motif (FLDD), and a Sensor 1-like motif (ITSNV). E2 contained a leucine zipper-like motif with heptad-spaced leucine residues.

In the late genes and regulatory region, L2 harbored multiple overlapping R-x-[KR]-R furin-like motifs near the C-terminus; however, no canonical N-terminal furin-cleavage site was detected. Additional L2 features included a glycine-rich G-x₃-G region in the N-terminal portion, a conserved FYL-like retromer-binding motif, and a C-terminal Lys/Arg-rich basic region. L1 similarly displayed a Lys/Arg-rich C-terminal basic tail. In the LCR/URR, one canonical E2-binding site, two E1 recognition hexanucleotide-like motifs, and one AATAAA polyA-like hexamer were identified, whereas additional AATAAA hexamers and non-LCR/URR E2BS-like matches are summarized in Supplementary Table S1. Conserved and putative functional motifs identified in CpPV1 proteins and in the LCR/URR are summarized in Table 1 and Supplementary Table S1 and illustrated in Supplementary Figures S1–S8.

**Table 1:** Predicted motifs identified in the CpPV1 genome, including consensus patterns, positions, and annotation comments

Gene/ region	Fragment size (nt)	Motif	Consensus/pattern	Position (interval)*	Instance/comment
E6	411	Zinc-binding	C-X2-C-X29-C-X2-C	aa 25–61; 98–134	Two conserved Cys-rich zinc-binding motifs detected
		PDZ-binding (class I)	[ST]-X-[ACVILFMWY]-L	—	Absent at the C-terminus
E7	282	Zinc-binding	C-X2-C-X29-C-X2-C	aa 50–86	Conserved Cys-rich zinc-binding motif detected
		pRb-binding	L-X-C-X-E	—	No canonical or close variants detected
E1	1803	Walker A (P-loop)	G-X4-G-K-[TS]	aa 430–437	Exact motif GPPDTGKS detected, consistent with the ATP-binding site of the E1 helicase
		Walker B-like motif	hhhhD[D/E]	aa 473–476	Putative Walker B-like motif (FLDD) detected within the E1 helicase domain
		Sensor 1-like motif	V/I-T-S-N-I/V	aa 517–521	Putative Sensor 1-like motif (ITSNV) detected within the E1 helicase domain
LCR/URR	—	E1 recognition hexanucleotide (E1BS-like)	AACAAT	nt 8722–8727; 8783–8788	Two exact matches detected in the terminal regulatory region
E2	1047	Leucine zipper-like motif	L-X6-L-X6-L	aa 4–18	Heptad-like leucine spacing detected
LCR/URR	—	E2BS	ACCN6GGT	nt 8676–8687	Exact match detected in the terminal regulatory region
L2	1560	Furin cleavage	R-X-[KR]-R	aa 508–515	Multiple overlapping exact matches detected near the C-terminus; no canonical N-terminal furin cleavage motif identified
		Putative N-terminal transmembrane-like motif	GxxxG-rich region	aa 57–67	Multiple overlapping G-X(3)-G motifs detected within the N-terminal glycine-rich segment
		polyA-like hexamer	AATAAA	nt 5308–5313	Exact AATAAA hexamer detected
		Putative C-terminal retromer-binding motif	FYL-like	aa 499–501	Conserved FYL motif detected near the C-terminus
		Putative C-terminal basic tail / NLS-like region	K/R-rich	aa 499–519	C-terminal Lys/Arg-rich region detected; strongest basic cluster at aa 506–519
L1	1533	Putative C-terminal NLS / DNA-binding basic tail	K/R-rich	aa 478–510	C-terminal Lys/Arg-rich tail detected; strongest basic cluster at aa 490–510
LCR/URR	—	polyA-like hexamer	AATAAA	nt 8428–8433	Exact AATAAA hexamer detected

Protein motifs are reported in amino acid coordinates (aa), whereas regulatory nucleotide motifs are reported in nucleotide coordinates (nt). Only exact matches on the annotated genomic strand were tabulated for DNA motifs

To contextualize CpPV1 within known rodent PV diversity, we compiled all rodent papillomaviruses with complete or reference genome records available in PaVE/GenBank, yielding 20 entries (Table 2). The dataset is taxonomically dominated by genus Pipapillomavirus (8/20) and Iotapapillomavirus (5/20), with single representatives of Sigmapapillomavirus and Dyosigmapapillomavirus, and five unclassified viruses. Detection contexts span a broad spectrum, including cutaneous proliferative lesions, pigmented plaques, oral and anogenital lesions, intestinal and lymphoid tissues, fecal and environmental samples, and metagenomic surveys not associated with specific lesions.

**Table 2:** Rodent papillomaviruses previously reported, with host, ICTV classification, GenBank/RefSeq accession, and isolation site/lesion

Host	Virus / Type	GenBank accession*	Genus	ICTV Species	Lesion/Isolation site
*Apodemus chevrieri*	AchePV1	KY370096 [17]	Pipapillomavirus	Unclassified	Not reported.
*Apodemus sylvaticus*	AsPV1	HQ625440 [18]	Pipapillomavirus	Pipapillomavirus 2	Normal ear skin (no visible lesion).
*Apodemus sylvaticus*	AsPV2	PQ576917 [19]	Unclassified	Unclassified	Fecal sample from small terrestrial mammals in Spain; not lesion-specific.
*Apodemus sylvaticus*	AsPV3	BK066393 [20]	Unclassified	Unclassified	Wood mouse metagenome / environmental sample; not lesion-specific.
*Castor canadensis*	CcanPV1	KC020689 [21]	Dyosigmapapillomavirus	Dyosigmapapillomavirus 1	Cutaneous proliferative lesions (feet, nose).
*Erethizon dorsatum*	EdPV1	AY684126 [22]	Sigmapapillomavirus	Sigmapapillomavirus 1	Benign cutaneous papillomatous lesions (footpads, facial skin).
*Erethizon dorsatum*	EdPV2	MH376689 [23]	Unclassified	Unclassified	Pigmented viral plaques.
*Marmota caudata*	McauPV1	PP728737	Unclassified	Unclassified	Detected in intestine and lymph nodes of long-tailed marmots; not lesion-specific.
*Mastomys coucha*	McPV2	DQ664501 [8]	Pipapillomavirus	Pipapillomavirus 2	Anogenital lesions/condylomas; cutaneous lesions also reported.
*Mastomys natalensis*	MnPV1	U01834 [24]	Iotapapillomavirus	Iotapapillomavirus 1	Cutaneous keratoacanthomas.
*Meriones unguiculatus*	MungPV1	BK066449 [20]	Iotapapillomavirus	Unclassified	Not reported.
*Mesocricetus auratus*	MaPV1	E15111 [25]	Pipapillomavirus	Pipapillomavirus 1	Oral cavity; tongue dysplasia.
*Micromys minutus*	MmiPV1	DQ269468 [26]	Pipapillomavirus	Pipapillomavirus 2	Cutaneous papillomatous lesions.
*Mus musculus*	MmuPV1	GU808564 [9]	Pipapillomavirus	Pipapillomavirus 2	Snout cutaneous papillomas; oral mucosa in experiments
*Peromyscus* maniculatus	PmPV1	JF755418 [27]	Iotapapillomavirus	Unclassified	Fecal sample / fecal viral flora of wild rodents; not lesion-specific.
*Phodopus sungorus*	PsuPV1	HG939559 [28]	Pipapillomavirus	Pipapillomavirus 1	Anogenital lesion; also detected in asymptomatic oral/mucosal swabs.
*Rattus norvegicus*	RnPV1	GQ180114 [29]	Pipapillomavirus	Pipapillomavirus 2	Oral mucosa (oral cavity).
*Rattus norvegicus*	RnPV2	HQ625441 [18]	Iotapapillomavirus	Iotapapillomavirus 1	Rectal smear; additional detection in facial hair follicles; no visible lesions.
*Rattus norvegicus*	RnPV3	KT945134 [30]	Iotapapillomavirus	Iotapapillomavirus 1	Urban wild rat metagenomic sample (not lesion-specific).
*Rattus norvegicus*	RnPV4	MH892425 [31]	Unclassified	Unclassified	Oral swab-derived isolate; not lesion-specific. This assignment is supported by the accession name and the wild-rat oral/skin/feces papillomavirus genome paper.

*GenBank accessions without associated publication reference are direct submission records

Among lesion-associated records, several rodent PVs including CcanPV1, EdPV1, EdPV2, MnPV1, MmiPV1, MmuPV1, and PsuPV1 have been linked to epithelial proliferative disease at cutaneous or mucocutaneous sites. Others were detected through non-targeted surveillance strategies rather than from overt lesions. Taken together, these records indicate that the known rodent PV repertoire encompasses both lesion-associated viruses and lineages identified opportunistically. Against this background, the detection of PV DNA in association with a histopathologically confirmed exophytic cutaneous papilloma in *C. paca* is consistent with the heterogeneous spectrum of rodent papillomavirus presentations documented to date.

### Pairwise L1 identities

**Fig. 6:**
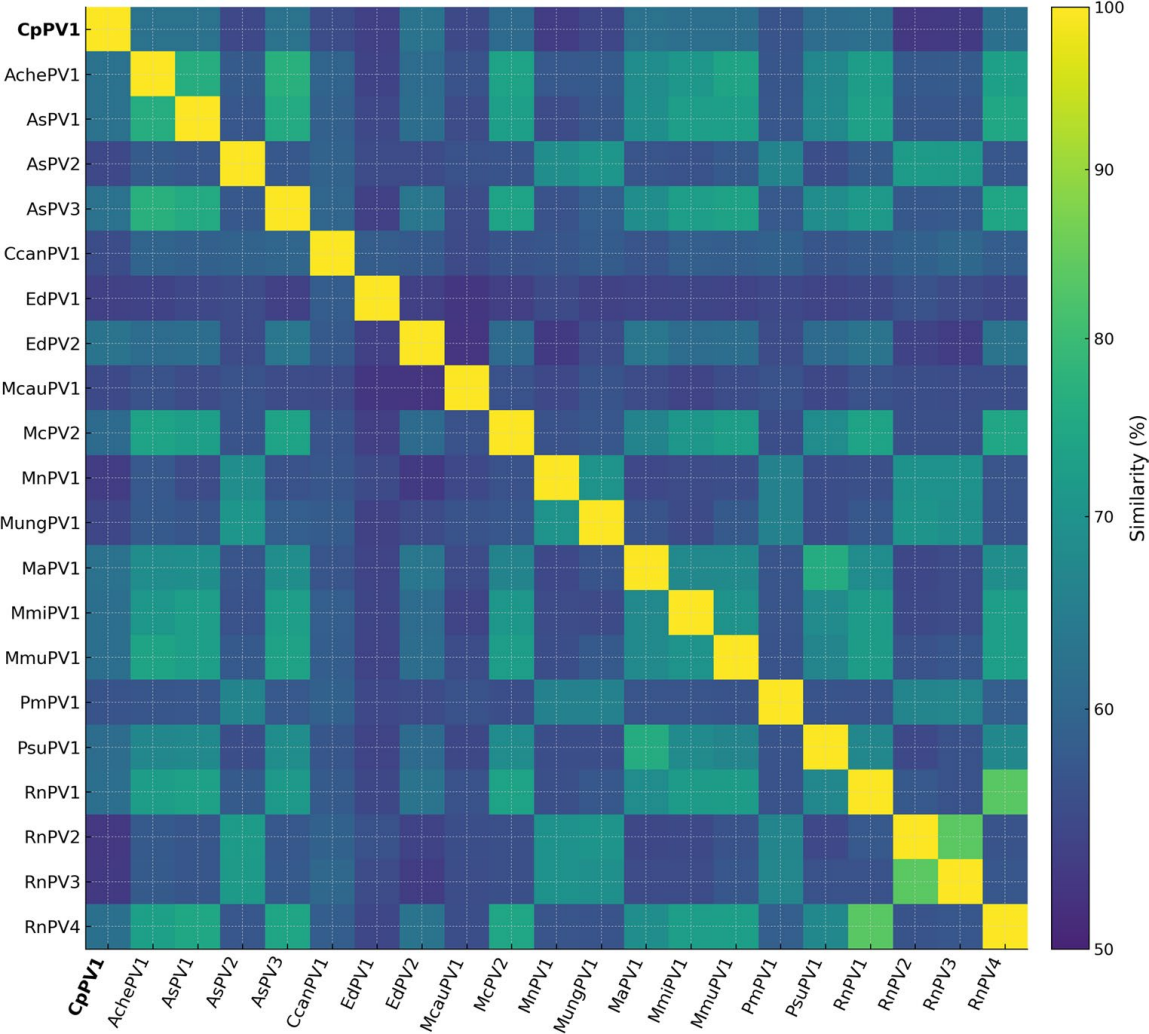
Heatmap of pairwise L1 nucleotide similarity (%) among rodent papillomaviruses based on the pairwise percent identity matrix obtained directly in Geneious Prime from a translation-guided MUSCLE alignment of complete L1 nucleotide sequences, followed by manual trimming (final alignment length: 1,494 nt). Virus labels are shown on both axes, with CpPV1 in bold to denote the novel paca papillomavirus characterized in this study

Pairwise nucleotide identities for the complete L1 gene showed that CpPV1 shared the highest L1 nucleotide identity with *Erethizon dorsatum* papillomavirus 2 (EdPV2) at 63.10%, making EdPV2 the closest currently known relative of CpPV1 among rodent PVs. Notably, EdPV2 was detected in a North American porcupine, another member of Hystricomorpha, reinforcing the relevance of this pairing. In contrast, EdPV2 shared only 53.61% L1 nucleotide identity with *Erethizon dorsatum* papillomavirus 1 (EdPV1), indicating that it does not group most closely with the other currently known papillomavirus from the same host species. Nevertheless, the CpPV1–EdPV2 value falls well below both the 90% threshold used for PV type demarcation and the 70% threshold commonly applied in species-level comparisons [11], unambiguously supporting CpPV1 as a distinct papillomavirus type. All other rodent PVs showed lower L1 similarity to CpPV1, indicating marked sequence divergence across the dataset (Fig. 6; Supplementary Table S2).

### Phylogenetic analysis

**Fig. 7:**
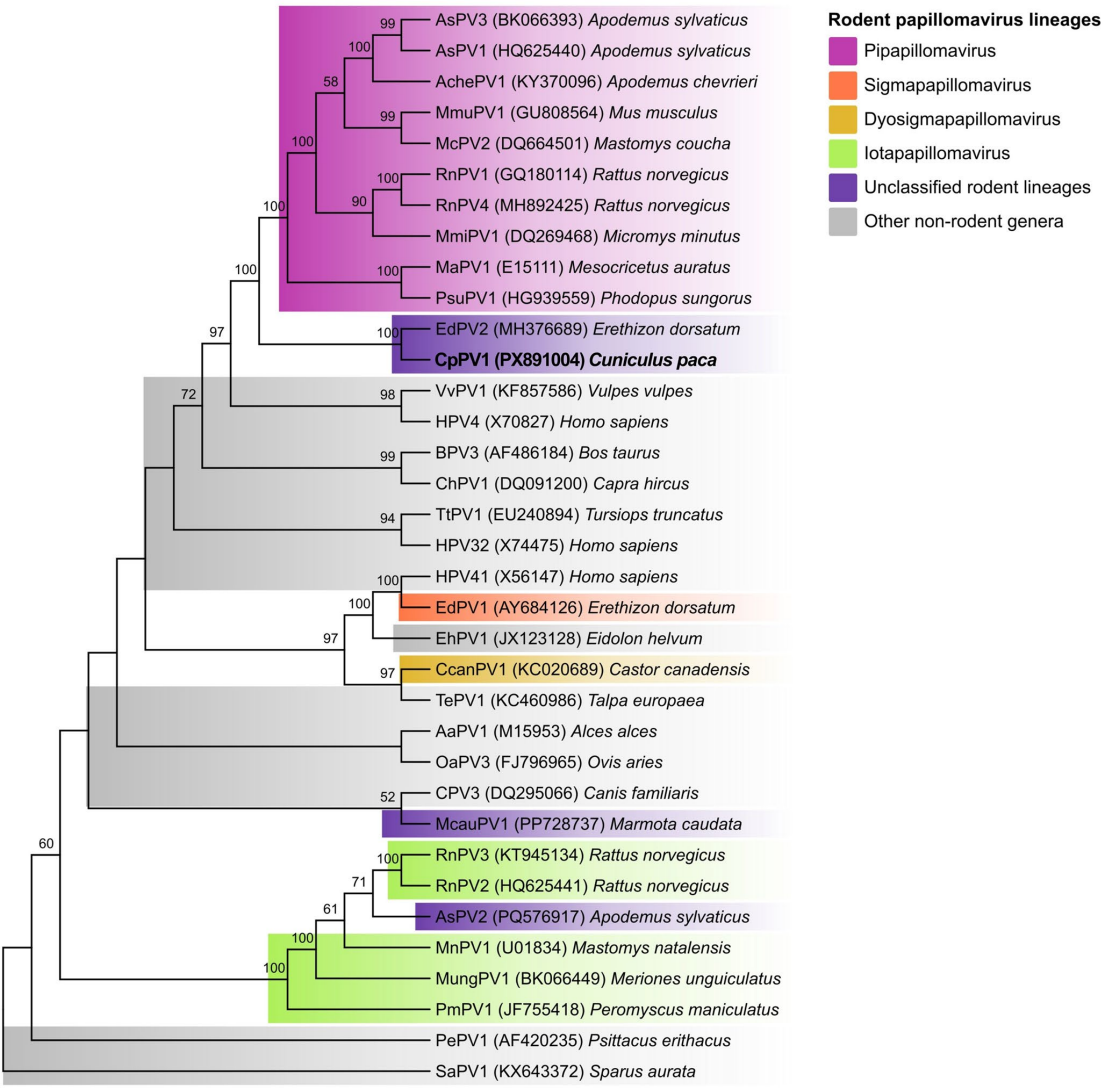
Maximum-likelihood phylogeny inferred from the concatenated E1–E2–L2–L1 nucleotide dataset. Colored boxes highlight the main rodent papillomavirus lineages recovered in the present analysis, whereas other non-rodent genera are shown in gray. CpPV1 is shown in bold. Bootstrap values ≥ 50 are indicated at the nodes. The tree recovers CpPV1 as the sister lineage of EdPV2 and shows that rodent papillomaviruses are distributed across multiple lineages rather than forming a single monophyletic group

Maximum-likelihood phylogenetic analysis of the concatenated E1–E2–L2–L1 nucleotide dataset recovered CpPV1 as the sister lineage of EdPV2 with strong bootstrap support (100; Fig. 7), a result fully congruent with the pairwise L1 identity analysis. Together, these findings confirm CpPV1 as a distinct PV type and place it within a well-supported unclassified rodent lineage currently represented by EdPV2.

The phylogeny further demonstrates that rodent papillomaviruses do not form a single monophyletic group within the family *Papillomaviridae*; instead, they are distributed across multiple lineages, including *Pipapillomavirus*, *Iotapapillomavirus*, *Sigmapapillomavirus*, *Dyosigmapapillomavirus*, and distinct unclassified clades. CpPV1 does not cluster with either of the two largest established rodent genera, but forms a well-supported clade exclusively with EdPV2, reinforcing its phylogenetically distinct placement. Although AsPV3 and RnPV4 remain unclassified in current source databases such as PaVE, GenBank, and ICTV, as summarized in Table 2, both grouped within the broader *Pipapillomavirus* cluster in the present phylogeny, consistent with their higher pairwise L1 similarities to members of that lineage. By contrast, AsPV2 and McauPV1 remained outside the established rodent genera recovered here. This pattern highlights the uneven taxonomic resolution of currently known rodent PV diversity and suggests that some presently unclassified viruses may nevertheless show stable affinity to recognized rodent PV radiations in phylogenetic analyses.

The association between CpPV1 and EdPV2 is also notable from a host perspective: both viruses were detected in Hystricomorpha rodents from the Americas (*C. paca* and *E. dorsatum*, respectively), and their genomes are nearly identical in size (8,803 bp and 8,809 bp, respectively). Considered together, the high relative affinity between CpPV1 and EdPV2, their exclusive grouping in the concatenated phylogeny, and the comparatively low L1 identity between EdPV2 and EdPV1 support the interpretation that the CpPV1–EdPV2 relationship reflects a distinct evolutionary lineage rather than a simple host-label association. While the present dataset is insufficient to formally infer co-evolutionary history, this pattern suggests that additional sampling within Hystricomorpha may uncover further papillomavirus diversity in this understudied host group. An expanded family-level phylogeny including the full papillomavirus backbone is provided in Supplementary Figure S1.

## Discussion

The characterization of CpPV1 fills a specific gap in the distribution of papillomavirus diversity across Rodentia. Until now, molecular PV data from South American hystricognath rodents were entirely absent, and the available rodent PV literature has been shaped almost exclusively by records from Europe, North America, and Asia [21, 26, 28, 29, 31]. The present findings therefore shift attention to a host group Neotropical hystricognaths that has been largely invisible in papillomavirus surveillance, and demonstrate that molecular screening of clinically apparent lesions in free-living animals can yield genomically complete and phylogenetically informative PV genomes even in resource-limited field settings.

From a clinicopathological perspective, the present case fits within the broader but heterogeneous spectrum of rodent papillomavirus-associated disease. The histopathological triad of acanthosis, orthokeratotic hyperkeratosis, and koilocytosis carries diagnostic weight beyond simple morphological description: koilocytosis in particular is a cytopathic hallmark of active papillomavirus infection reflecting viral-induced perinuclear vacuolization, and its presence alongside epithelial hyperplasia strengthens the biological plausibility of the CpPV1–lesion association [32, 33]. This distinction matters because not all rodent PV detections have a histopathologically confirmed lesion counterpart several entries in Table 2 derive from metagenomic surveys, fecal samples, or swabs without associated pathology making the present case one of the better-documented examples of a lesion-associated rodent PV outside the established genera. When compared with the lesion types summarized in Table 2, the cutaneous papilloma observed here is most consistent with those reported for viruses phylogenetically close to CpPV1: EdPV1 and EdPV2 in porcupines (*Erethizon dorsatum*) and MmiPV1 in *Micromys minutus*, all associated with exophytic cutaneous proliferations [22, 23, 26].

In contrast, members of *Iotapapillomavirus* the most extensively studied rodent PV genus, exemplified by MmuPV1 in *Mus musculus* have been associated not only with benign cutaneous papillomas but also with mucosal disease and progression to squamous cell carcinoma, particularly under immunosuppression, illustrating a broader pathogenic range than what is currently documented for the CpPV1–EdPV2 lineage [3, 4]. At the same time, the rodent papillomavirus literature also includes viruses detected in oral, rectal, anogenital, or clinically unapparent contexts, as documented for *Rattus norvegicus* and *Phodopus sungorus* PVs [18, 28, 29]. Taken together, these comparisons indicate that CpPV1 is best interpreted as part of the lesion-associated end of a broader and biologically diverse rodent PV spectrum summarized in Table 2.

Although only a single lesion was evaluated, its localized and apparently benign presentation is more compatible with a limited cutaneous infection than with evidence of substantial disease burden in this host species. As the lesion was detected during examination and removed immediately, no inference can be made about duration, recurrence, or clinical progression. This limitation is important, because papillomavirus-associated outcomes in rodents are clearly variable across lineages and host conditions. The oncogenic potential of PVs is nonetheless relevant to acknowledge in this context: the viral oncoproteins E6 and E7 interfere with the tumor suppressor proteins p53 and pRb, respectively, and their divergent motif architecture in CpPV1 including the absence of a canonical LxCxE motif in E7 and the lack of a PDZ-binding motif in E6 represents an open question for future functional investigation [2, 6]. The present study supports the interpretation that CpPV1 is associated with a cutaneous papilloma in *C. paca*, but does not by itself allow conclusions about prevalence, pathogenicity at the population level, or long-term clinical relevance in this host.

The genomic architecture of CpPV1 places it unambiguously within *Papillomaviridae*. The absence of an E5 ORF is worth contextualizing: E5 is variably present across PV lineages and its loss in rodent PVs is not unusual, suggesting that E5-mediated mechanisms well characterized in human and bovine papillomaviruses, where E5 modulates growth factor receptor activity are not a conserved feature of this host–virus group [11]. More informative than the presence or absence of individual ORFs is the motif-level divergence within otherwise conserved proteins. The lack of a canonical PDZ-binding motif in E6, a canonical LxCxE motif in E7, and a canonical N-terminal furin-cleavage site in L2 is not easily interpreted as functional loss in isolation rather, it suggests that CpPV1 may engage host cell machinery through alternative or divergent molecular interfaces, a pattern increasingly recognized among non-human PVs with limited functional characterization [6, 34–37]. Resolving whether these divergences have functional consequences will require experimental approaches beyond genome characterization. The use of RCA followed by HTS and de novo assembly is in line with validated approaches for recovering circular DNA virus genomes [15, 18], and the assembly metrics 13,994 supporting reads at a mean coverage of 272.9× provide confidence in the completeness and accuracy of the recovered genome.

The phylogenetic signal in the concatenated E1–E2–L2–L1 dataset is unambiguous on two points that carry different interpretive weight. The first that CpPV1 is a distinct type is unsurprising given the sequence divergence observed in the FAP-based screening, and the 63.10% L1 nucleotide pairwise identity to EdPV2 confirms this with a value far below the 90% L1 nucleotide identity threshold used for papillomavirus type demarcation [11]. Moreover, because this value also falls below the 70% L1 nucleotide identity threshold used for papillomavirus species demarcation [11], CpPV1 is best regarded as clearly distinct from EdPV2 at the species level as well. The second and more consequential point is the consistent and strongly supported placement of CpPV1 and EdPV2 in the same exclusive phylogenetic clade, distinct from the established *Pipapillomavirus* and *Iotapapillomavirus* lineages. This interpretation is not altered by the heterogeneous placement of currently unclassified rodent PVs in the present analysis: although AsPV3 and RnPV4 grouped within the broader *Pipapillomavirus* cluster, CpPV1 and EdPV2 remained outside the established rodent genera and formed a separate, well-supported lineage. This is not trivial it means the CpPV1–EdPV2 relationship reflects a genuine evolutionary affinity between two geographically and taxonomically separated hystricognath-infecting lineages, rather than a simple consequence of shared host order.

The relationship between CpPV1 and EdPV2 is further strengthened by genomic similarity beyond L1 identity alone. EdPV2 was originally highlighted as exceptional among rodent papillomaviruses because of its unusually large genome [23], and CpPV1 shares a nearly identical genome size (8,803 bp versus 8,809 bp), a feature that further distinguishes this lineage from most other characterized rodent PVs and raises questions about whether genome expansion is a shared derived character of this clade. This interpretation is reinforced by the pairwise L1 results: EdPV2 shared higher nucleotide identity with CpPV1 than with EdPV1, the other currently known porcupine papillomavirus, indicating that the CpPV1–EdPV2 affinity is not simply explained by host identity alone. It is also worth noting that a divergent genomic variant of EdPV2 (GenBank MZ647949) sharing only approximately 93% identity with the reference sequence (MH376689) has been described, indicating that intra-lineage diversity within this group may be greater than currently appreciated [46, 47]. Together with CpPV1, this suggests that the CpPV1–EdPV2 lineage is more heterogeneous than initially recognized and remains substantially under-sampled. Given that EdPV2 has long lacked formal genus assignment, the addition of CpPV1 as a second, phylogenetically distinct member of this clade strengthens the view that these viruses represent an emerging lineage within *Papillomaviridae* that may warrant formal taxonomic evaluation as additional genomes become available.

The CpPV1–EdPV2 association is also noteworthy from a host perspective. Both viruses were identified in hystricognath rodents from the Americas, namely *C. paca* and *E. dorsatum*, whereas the best-sampled rodent papillomavirus genera are dominated by non-hystricognath hosts such as *Mus musculus*, *Rattus norvegicus*, and *Micromys minutus*. Within New World hystricognaths, caviomorphs (the group to which *C. paca* belongs) and erethizontoids (to which *E. dorsatum* belongs) are related but phylogenetically distinct lineages whose divergence predates the separation of myomorph rodents from other rodent lineages [38, 39]. The clustering of CpPV1 and EdPV2 to the exclusion of non-hystricognath rodent PVs is therefore consistent with virus–host co-evolutionary patterns that have been well documented in *Papillomaviridae*, where viral phylogeny frequently mirrors host phylogeny at deeper taxonomic levels [11, 40]. CpPV1 thus provides preliminary evidence of host-associated papillomavirus diversification within Hystricognathi and calls for targeted sampling of additional species across both caviomorphs and erethizontoids to formally test whether this pattern reflects deeper co-divergence.

The broader ecological relevance of this finding lies primarily in biodiversity surveillance rather than zoonotic concern. Papillomaviruses are generally considered highly host-adapted, but under-sampled regions continue to reveal unexpected lineage diversity once molecular tools are applied [1, 10, 11]. Recent studies from the western Brazilian Amazon are particularly relevant in this context. In domestic dogs from Acre and Rondônia, CPV1 predominated and CPV8 was also detected, demonstrating the feasibility of papillomavirus field genomics in the region and revealing active viral circulation in managed animal populations at the wildlife–domestic interface [41]. In cattle from the same biome, integrated clinicopathological and molecular surveys have documented diverse BPV types and lesion morphologies, reinforcing that multiple PV lineages co-circulate across host species in this eco-epidemiological setting [42]. The detection of CpPV1 in a free-living paca from the same region adds a wild mammal host to this emerging picture, suggesting that the Amazonian papillomavirus landscape extends across the wildlife–livestock–companion animal continuum and underscores the value of including wildlife species in regional PV surveillance frameworks.

The broader context of papillomavirus research in human medicine offers a useful comparison at the level of surveillance logic. HPV-related disease remains a major global health burden, and marked regional disparities in baseline epidemiological data and prevention capacity have been documented even within underrepresented settings, including central Asia [43]. Although animal PVs have no zoonotic potential, the contrast between relatively well-developed HPV surveillance infrastructures in some regions and the near-complete absence of baseline PV data for wildlife in Neotropical systems reflects a shared underlying challenge: viral diversity cannot be managed or interpreted without prior characterization. Wildlife virological monitoring in biodiverse areas such as the Amazon can therefore complement broader papillomavirus research agendas by expanding the evolutionary and ecological framework within which human and animal PV diversity is understood.

The ecological profile of *C. paca* also makes this host particularly relevant for future surveillance efforts. As a widely distributed Neotropical game species present in both wild and managed settings, it occupies interfaces that are ecologically important and logistically accessible for monitoring [44, 45]. Given the persistent gaps in rodent papillomavirus surveillance in South America, the present study highlights the need for broader virome investigations targeting wild rodents in the Amazon, ideally integrating field ecology, veterinary pathology, genomics, and comparative evolutionary analysis.

In summary, this study provides the first molecular characterization of a papillomavirus infecting *C. paca*. The combined histopathological, genomic, and phylogenetic evidence supports CpPV1 as a distinct papillomavirus type. Additionally, it supports the existence of a hystricognath-associated papillomavirus lineage in the Americas whose taxonomy, genomic distinctiveness, and potential for co-diversification with host lineages warrant dedicated investigation. More broadly, this work expands the currently known diversity of *Papillomaviridae* in Neotropical rodents and supports continued surveillance of viral lineages circulating in tropical wildlife.

## Conclusion

This study broadens the still limited understanding of papillomavirus diversity in wild Neotropical rodents by documenting a lesion-associated papillomavirus in *Cuniculus paca*. Taken together, the histopathological, genomic, and evolutionary evidence supports CpPV1 as a distinct papillomavirus type and indicates that, together with EdPV2, it belongs to an emerging lineage of likely taxonomic significance within *Papillomaviridae*. The consistent grouping of CpPV1 with a porcupine-infecting PV from North America further suggests that hystricognath rodents from the Americas may harbor an underexplored and co-diversified PV lineage. More broadly, these findings highlight the importance of targeted wildlife surveillance in biodiverse and still under-sampled regions such as the Amazon for improving baseline knowledge of viral diversity and host associations.

## Supplementary Information

Below is the link to the electronic supplementary material.

Supplementary Material 3

## Data Availability

The complete genome sequence of CpPV1 has been deposited in GenBank under accession number PX891004. Rodent papillomavirus sequences used for comparative analyses are available in GenBank/RefSeq under the accessions listed in Table 2. Supplementary materials, including Supplementary Table S1 and Supplementary Figures S1–S8, are provided with this article. All other relevant data are contained within the article and its supplementary files.
