# Supplementary material for: Complete genome and phylogenetic characterization of a novel papillomavirus from Cuniculus paca in the Brazilian Amazon
Source: Arch Virol. 2026 May 7;171(6):172. doi: 10.1007/s00705-026-06636-y (PMC13152935; doi:10.1007/s00705-026-06636-y)

**Supplementary Material**

Supplementary Table S1. Additional motifs detected in the genome but not included in the main table

| **Gene/region** | **Fragment size (nt)** | **Motif** | **Consensus/pattern** | **Position (interval)*** | **Instance/comment** |
| --- | --- | --- | --- | --- | --- |
| Genome (outside LCR/URR) | — | Additional E2BS-like matches | ACCN6GGT | nt 2950–2961; 5541–5552 | Two additional exact matches detected outside the terminal regulatory region; only the LCR/URR-associated E2BS was retained in the main table. |
| E1/internal region | 1803 | Additional polyA-like hexamer | AATAAA | nt 2356–2361 | Exact AATAAA hexamer detected outside the main polyadenylation contexts prioritized in the main table. |
| Internal noncoding region | — | Additional polyA-like hexamers | AATAAA | nt 3573–3578; 3730–3735 | Two additional exact AATAAA hexamers detected outside the LCR/URR-associated site retained in the main table. |
| L2 | 1560 | Putative SNX17-binding motifs | NPxF/Y | aa 192–195; 288–291;  303–306 | Three exact NPxF-type motifs detected in L2 (NPSF, NPAF, and NPAF); retained in the supplementary table because their functional relevance in this genome remains untested. |

*Note: Protein motifs are reported in amino acid coordinates (aa), whereas regulatory nucleotide motifs are reported in nucleotide coordinates (nt). Only exact matches on the annotated genomic strand were tabulated for DNA motifs.

Supplementary Table S2. Pairwise L1 nucleotide identity (%) matrix among rodent papillomaviruses included in the comparative dataset.

| **Virus / type** | **CpPV1** | **AchePV1** | **AsPV1** | **AsPV2** | **AsPV3** | **CcanPV1** | **EdPV1** | **EdPV2** | **McauPV1** | **McPV2** | **MnPV1** | **MungPV1** | **MaPV1** | **MmiPV1** | **MmuPV1** | **PmPV1** | **PsuPV1** | **RnPV1** | **RnPV2** | **RnPV3** | **RnPV4** |  |
| --- | --- | --- | --- | --- | --- | --- | --- | --- | --- | --- | --- | --- | --- | --- | --- | --- | --- | --- | --- | --- | --- | --- |
| **CpPV1** |  | 62,72 | 62,38 | 54,75 | 62,52 | 55,49 | 53,59 | 63,10 | 54,93 | 61,24 | 53,08 | 54,35 | 62,57 | 61,90 | 61,98 | 56,49 | 61,49 | 61,98 | 52,41 | 52,54 | 62,12 |  |
| **AchePV1** | 62,72 |  | 76,44 | 57,97 | 76,97 | 59,77 | 53,95 | 61,31 | 56,34 | 73,96 | 57,63 | 57,83 | 68,88 | 70,96 | 74,10 | 56,83 | 67,27 | 72,36 | 57,70 | 57,90 | 73,16 |  |
| **AsPV1** | 62,38 | 76,44 |  | 57,23 | 75,70 | 59,17 | 54,69 | 61,71 | 55,15 | 73,03 | 55,42 | 56,89 | 69,15 | 72,56 | 72,96 | 57,30 | 67,81 | 73,49 | 56,96 | 57,03 | 74,63 |  |
| **AsPV2** | 54,75 | 57,97 | 57,23 |  | 57,36 | 59,44 | 55,35 | 55,15 | 56,22 | 56,63 | 68,95 | 70,63 | 56,72 | 56,16 | 57,76 | 66,67 | 55,65 | 57,90 | 72,11 | 71,91 | 57,23 |  |
| **AsPV3** | 62,52 | 76,97 | 75,70 | 57,36 |  | 59,97 | 53,48 | 63,52 | 55,42 | 73,76 | 56,16 | 58,70 | 68,95 | 72,69 | 73,69 | 57,56 | 68,75 | 71,35 | 57,43 | 57,83 | 74,23 |  |
| **CcanPV1** | 55,49 | 59,77 | 59,17 | 59,44 | 59,97 |  | 58,30 | 57,76 | 55,02 | 56,49 | 57,10 | 58,17 | 56,72 | 58,70 | 58,37 | 59,37 | 57,12 | 57,90 | 59,37 | 60,37 | 58,37 |  |
| **EdPV1** | 53,59 | 53,95 | 54,69 | 55,35 | 53,48 | 58,30 |  | 53,61 | 52,34 | 53,28 | 55,06 | 53,82 | 54,03 | 54,22 | 54,08 | 54,80 | 54,17 | 54,55 | 56,16 | 55,22 | 54,42 |  |
| **EdPV2** | 63,10 | 61,31 | 61,71 | 55,15 | 63,52 | 57,76 | 53,61 |  | 52,07 | 61,11 | 52,54 | 55,15 | 63,64 | 61,38 | 61,71 | 55,29 | 61,16 | 62,99 | 53,88 | 53,08 | 62,99 |  |
| **McauPV1** | 54,93 | 56,34 | 55,15 | 56,22 | 55,42 | 55,02 | 52,34 | 52,07 |  | 56,22 | 54,69 | 56,63 | 55,24 | 54,17 | 55,62 | 56,29 | 54,50 | 56,22 | 55,76 | 55,49 | 55,69 |  |
| **McPV2** | 61,24 | 73,96 | 73,03 | 56,63 | 73,76 | 56,49 | 53,28 | 61,11 | 56,22 |  | 56,22 | 57,23 | 66,47 | 70,95 | 72,89 | 55,76 | 68,48 | 73,83 | 55,82 | 55,89 | 74,56 |  |
| **MnPV1** | 53,08 | 57,63 | 55,42 | 68,95 | 56,16 | 57,10 | 55,06 | 52,54 | 54,69 | 56,22 |  | 70,01 | 54,70 | 55,22 | 55,69 | 65,99 | 55,71 | 55,82 | 69,75 | 69,88 | 56,69 |  |
| **MungPV1** | 54,35 | 57,83 | 56,89 | 70,63 | 58,70 | 58,17 | 53,82 | 55,15 | 56,63 | 57,23 | 70,01 |  | 56,59 | 55,06 | 57,76 | 66,06 | 55,65 | 57,36 | 70,36 | 69,42 | 56,63 |  |
| **MaPV1** | 62,57 | 68,88 | 69,15 | 56,72 | 68,95 | 56,72 | 54,03 | 63,64 | 55,24 | 66,47 | 54,70 | 56,59 |  | 67,61 | 67,94 | 56,72 | 76,08 | 68,41 | 54,50 | 55,17 | 68,95 |  |
| **MmiPV1** | 61,90 | 70,96 | 72,56 | 56,16 | 72,69 | 58,70 | 54,22 | 61,38 | 54,17 | 70,95 | 55,22 | 55,06 | 67,61 |  | 70,01 | 56,76 | 68,28 | 72,09 | 54,82 | 55,15 | 72,62 |  |
| **MmuPV1** | 61,98 | 74,10 | 72,96 | 57,76 | 73,69 | 58,37 | 54,08 | 61,71 | 55,62 | 72,89 | 55,69 | 57,76 | 67,94 | 70,01 |  | 56,56 | 66,80 | 72,29 | 56,36 | 57,30 | 73,09 |  |
| **PmPV1** | 56,49 | 56,83 | 57,30 | 66,67 | 57,56 | 59,37 | 54,80 | 55,29 | 56,29 | 55,76 | 65,99 | 66,06 | 56,72 | 56,76 | 56,56 |  | 56,59 | 56,36 | 66,80 | 67,00 | 58,63 |  |
| **PsuPV1** | 61,49 | 67,27 | 67,81 | 55,65 | 68,75 | 57,12 | 54,17 | 61,16 | 54,50 | 68,48 | 55,71 | 55,65 | 76,08 | 68,28 | 66,80 | 56,59 |  | 67,27 | 54,70 | 56,25 | 67,47 |  |
| **RnPV1** | 61,98 | 72,36 | 73,49 | 57,90 | 71,35 | 57,90 | 54,55 | 62,99 | 56,22 | 73,83 | 55,82 | 57,36 | 68,41 | 72,09 | 72,29 | 56,36 | 67,27 |  | 57,50 | 56,29 | 83,87 |  |
| **RnPV2** | 52,41 | 57,70 | 56,96 | 72,11 | 57,43 | 59,37 | 56,16 | 53,88 | 55,76 | 55,82 | 69,75 | 70,36 | 54,50 | 54,82 | 56,36 | 66,80 | 54,70 | 57,50 |  | 84,11 | 56,63 |  |
| **RnPV3** | 52,54 | 57,90 | 57,03 | 71,91 | 57,83 | 60,37 | 55,22 | 53,08 | 55,49 | 55,89 | 69,88 | 69,42 | 55,17 | 55,15 | 57,30 | 67,00 | 56,25 | 56,29 | 84,11 |  | 57,23 |  |
| **RnPV4** | 62,12 | 73,16 | 74,63 | 57,23 | 74,23 | 58,37 | 54,42 | 62,99 | 55,69 | 74,56 | 56,69 | 56,63 | 68,95 | 72,62 | 73,09 | 58,63 | 67,47 | 83,87 | 56,63 | 57,23 |  |  |

**Note:** Pairwise nucleotide identity values were calculated from the manually trimmed, translation-guided MUSCLE alignment of complete L1 nucleotide sequences (final alignment length: 1,494 nt).

Supplementary Figure S1. Family-level maximum-likelihood phylogeny of *Papillomaviridae* based on concatenated E1–E2–L2–L1 nucleotide sequences, highlighting rodent papillomavirus lineages and the phylogenetic position of CpPV1


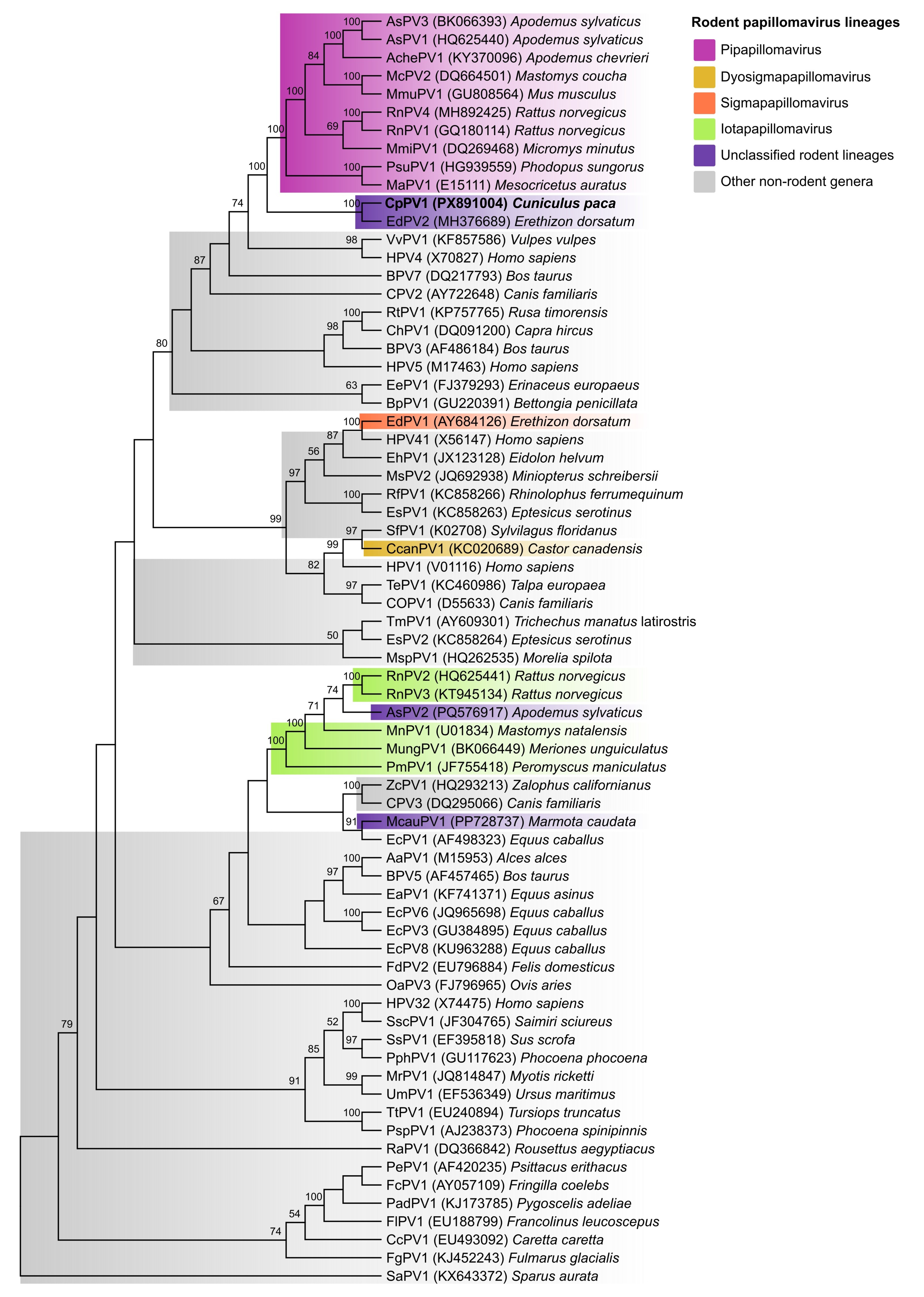


Supplementary Figure S2. Annotated E1 protein sequence of CpPV1 showing the Walker A, Walker B-like, and Sensor 1-like helicase motifs


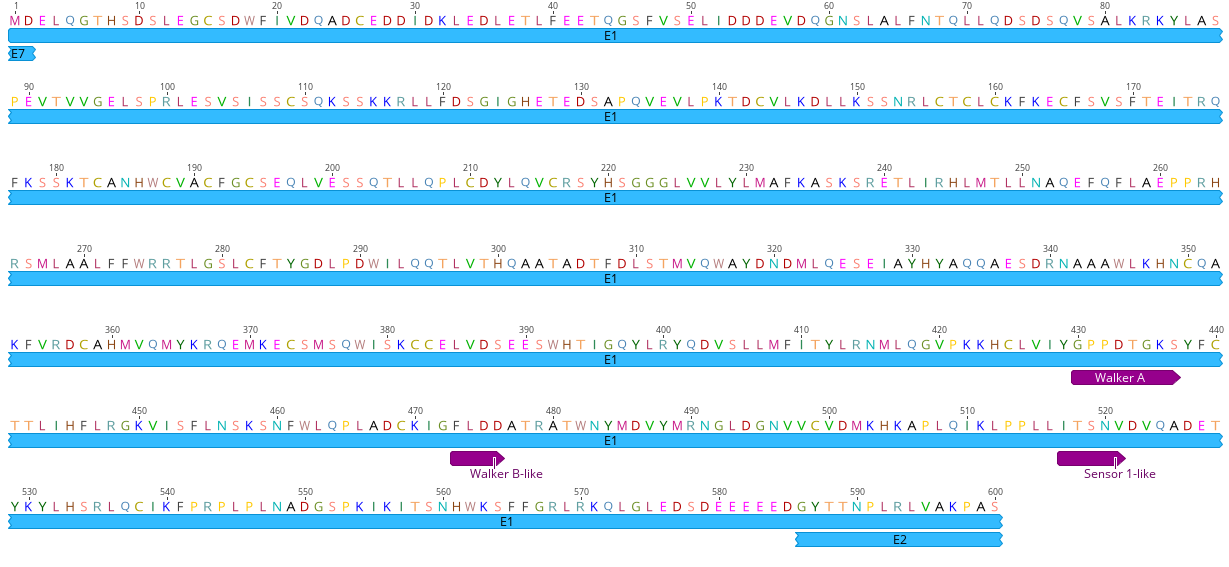


Supplementary Figure S3. Annotated E2 protein sequence of CpPV1 showing the N-terminal leucine zipper-like motif


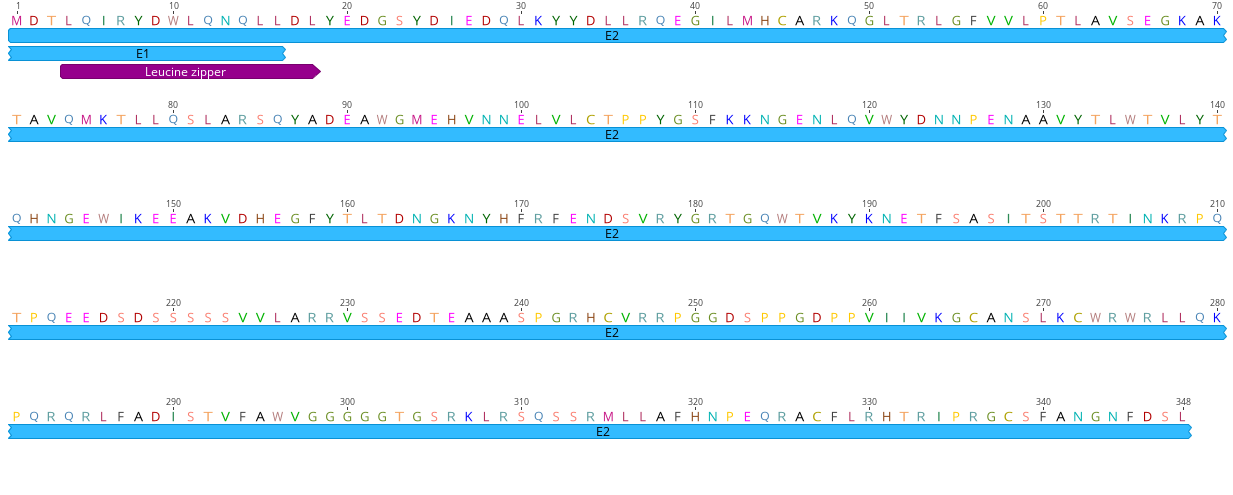


Supplementary Figure S4. Annotated E6 protein sequence of CpPV1 showing the two Cys-rich zinc-binding motifs


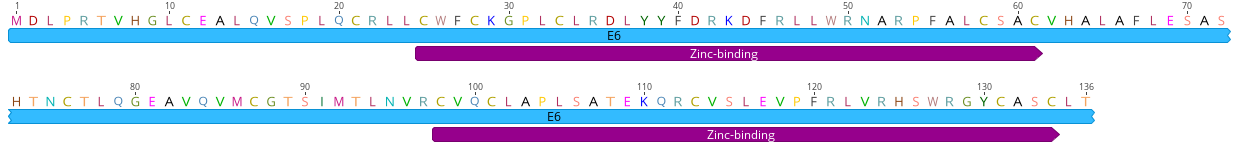


Supplementary Figure S5. Annotated E7 protein sequence of CpPV1 showing the conserved Cys-rich zinc-binding motif


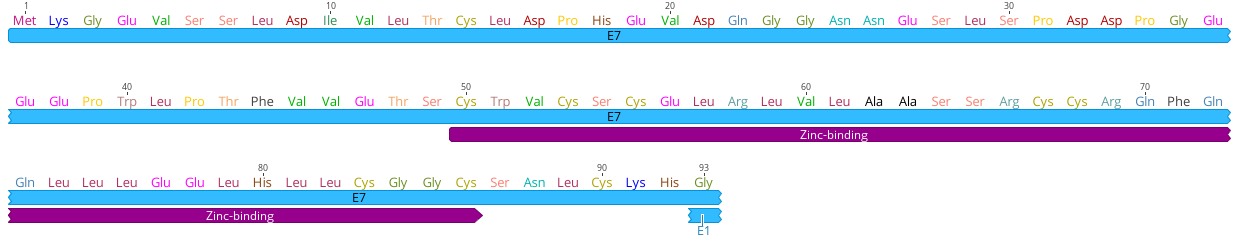


Supplementary Figure S6. Annotated partial L1 protein sequence of CpPV1 showing the C-terminal Lys/Arg-rich basic tail


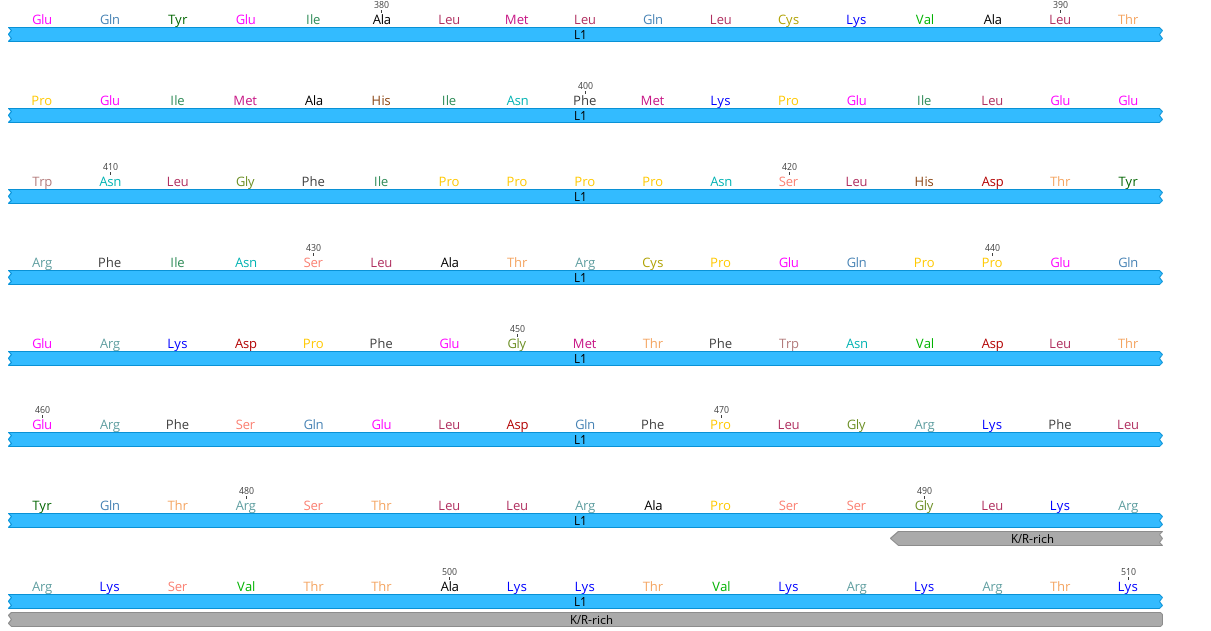


Supplementary Figure S7. Annotated L2 protein sequence of CpPV1 showing the positions of putative functional motifs


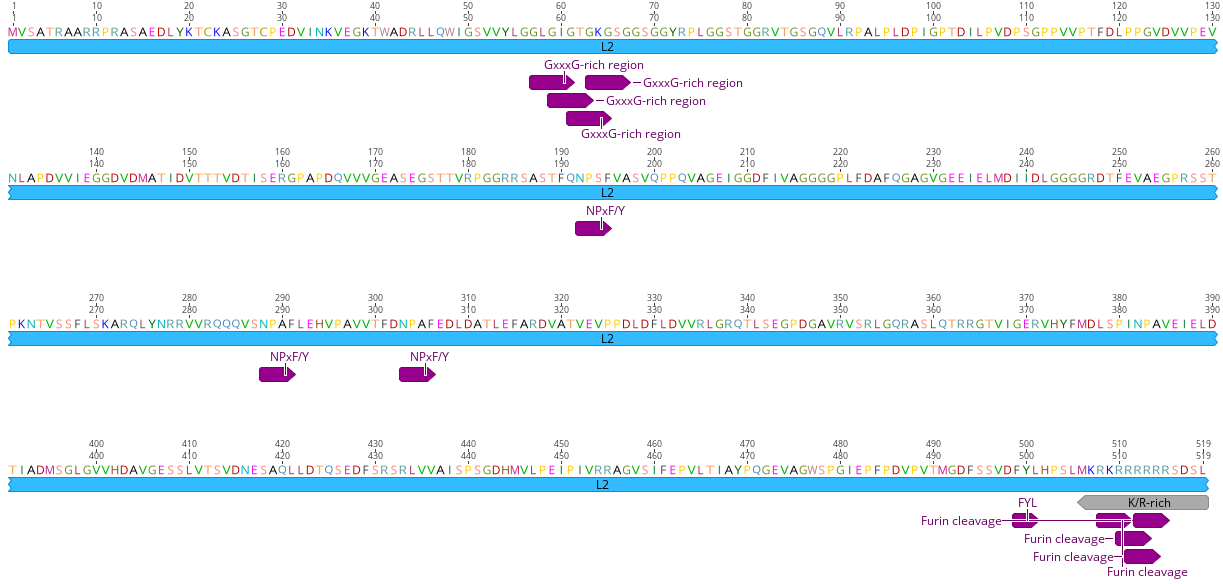


Supplementary Figure S8. Annotated LCR/URR nucleotide sequence of CpPV1 showing the E1 recognition hexanucleotides, E2BS, and polyadenylation-associated hexamer


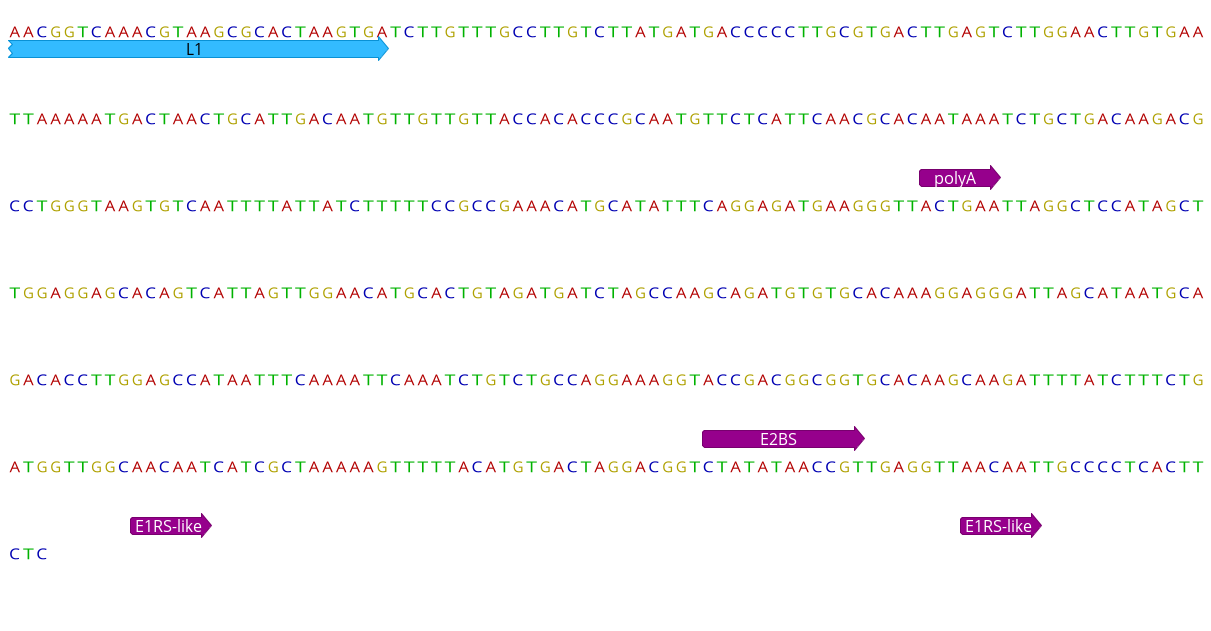

Supplement: Supplementary file 1 — Supplementary Material 3 [file 705_2026_6636_MOESM1_ESM.docx]
